# Citrullination of Collagen Type II and Epitope-Specific Antibody Responses in Rheumatoid Arthritis and Osteoarthritis: A Comparative Analysis

**DOI:** 10.3390/biology15201877

**Published:** 2026-10-18

**Authors:** Georgi Kalev, Stela Stoyanova, Borislav Mateev, Desislava Apostolova, Dzhemal Moten, Ivanka Teneva, Balik Dzhambazov, Tsvetelina Batsalova

**Affiliations:** Faculty of Biology, Paisii Hilensdarski University of Plovdiv, 4000 Plovdiv, Bulgaria; g.kalev@uni-plovdiv.bg (G.K.); stela.stoyanova@uni-plovdiv.bg (S.S.); mateev@uni-plovdiv.bg (B.M.); apostolova@uni-plovdiv.bg (D.A.); moten@uni-plovdiv.bg (D.M.); teneva@uni-plovdiv.bg (I.T.); balik@uni-plovdiv.bg (B.D.)

**Keywords:** citrullination, collagen type II, hyaline cartilage, osteoarthritis, rheumatoid arthritis

## Abstract

Citrullination of various proteins including the major structural protein of articular cartilage, collagen type II (COL2), has been indicated to play a role in rheumatoid arthritis (RA). Humoral responses against citrullinated COL2 epitopes have also been examined in patients with another common arthritic disorder, osteoarthritis (OA), but have not been shown to be present in a substantial proportion of patients in the studied groups. To further investigate this issue, we assessed anti-COL2 epitope responses in patients with OA and RA, with particular focus on immune responses to the citrullinated form of one of the main COL2 epitopes associated with RA. This research was combined with experiments aiming to define the involvement of COL2 citrullination in these two prevalent arthritic disorders by evaluation of the presence of citrulline in normal hyaline cartilage and in purified joint COL2 from RA and OA patients. Enzyme-linked immunosorbent assays (ELISA) were performed to determine the levels of antibodies specific to COL2 epitopes (C1, U1, J1 and citrullinated C1 epitope) in blood plasma of RA patients, OA patients and control individuals that do not suffer from arthritis disorder. The relative level of citrullination of COL2 purified from normal (healthy), OA and RA hyaline cartilage was assessed by direct ELISA. Immunofluorescent staining with anti-citrulline antibody was performed on histological sections of normal cartilage. Our results demonstrate significant reactivity to COL2 and three main COL2 epitopes, including citrullinated form of COL2 (358–369) epitope, in patients with OA. Collectively, more than 90% of the patients in the OA cohort showed reactivity against COL2 (358–369) epitope and/or citrullinated COL2 (358–369), which has not been observed in previous studies. A novel finding regarding COL2 citrullination of the present study is the determined tendency for higher relative level of citrullination of COL2 from arthritic patients compared to COL2 samples from individuals that do not suffer arthritic disorder. A tendency for increased relative anti-citrulline immunoreactivity for blood plasma proteins of RA patients compared to control individuals and OA patients was detected. Positive anti-citrulline staining was observed in the perichondrium and in the extracellular matrix surrounding chondrocytes in normal cartilage. Overall, our results indicate that citrullination is a physiological process that may become altered or enhanced during joint disease leading to bone destruction.

## Simple Summary

The present study involves evaluation of humoral responses against different collagen type II (COL2) epitopes in blood plasma of rheumatoid arthritis (RA) and osteoarthritis (OA) patients combined with investigation of the level of citrullination in normal hyaline cartilage, in joint COL2 and blood plasma during development of arthritic disorder that affects bone structure. It demonstrates that significant antibody reactivity against COL2 epitopes is present not only in RA but also in OA patients. Particularly, the OA patient group showed significant reactivity against a citrullinated COL2 epitope indicating possible involvement of immune response against citrullinated cartilage proteins in OA pathogenesis. Furthermore, our study demonstrates that citrulline amino acid residues are naturally present in healthy hyaline cartilage and provides preliminary numerical observations for increased relative citrullination level of joint COL2 in OA and RA patients, suggesting dysregulated citrullination dynamics during arthritic diseases. Development of two of the most common arthritic disorders could be associated with increased citrullination of joint COL2.

## 1. Introduction

More than 600 million people worldwide are affected by the two most common forms of arthritis—osteoarthritis (OA) and rheumatoid arthritis (RA). These diseases are also among the most common causes of chronic pain, which significantly impairs quality of life, leads to varying degrees of disability, and represents a substantial health and economic burden [1]. Currently, there is no cure for either OA or RA. Existing medications and therapeutic approaches aim to reduce pain and symptoms, as well as to improve the functional capacity of the affected joints [2].

Rheumatoid arthritis (RA) is defined as a chronic, systemic inflammatory disease of autoimmune origin that affects approximately 1% of the global population [3]. Although RA can affect individuals of any age, its incidence is highest among those aged 50–59 years [4]. The chronic inflammation of the synovial tissue characteristic of RA leads to the formation of pannus—an invasive tissue that causes destruction of the articular cartilage, erosion of the subchondral bone, and damage to the adjacent soft tissues [5]. Persistent inflammation and progressive destruction of cartilage and bone tissue ultimately result in functional disability and may contribute to increased mortality [3,5,6,7].

The etiology of RA remains incompletely understood, but interactions between the immune system, environmental factors, and genetic predisposition are considered to play a critical role in disease development [8]. Dysregulation of both innate and adaptive immunity leads to a loss of immune tolerance to self-structures. Consequently, an autoimmune response develops, characterized by the production of autoantibodies against various autoantigens [3,7]. The autoimmune process begins long before the onset of clinical symptoms, and RA-specific autoantibodies, such as rheumatoid factor (RF) and anti-citrullinated protein antibodies (ACPAs), can be detected years before the clinical manifestation of the disease [9,10].

Osteoarthritis is a heterogeneous disease characterized by structural changes throughout the entire joint, including cartilage degeneration, synovial membrane inflammation, joint stiffness, subchondral bone sclerosis, and periarticular muscle weakness, all of which contribute to joint instability and functional limitation [1]. Although OA has long been regarded as a disease associated with joint aging, increasing evidence indicates that its pathogenesis involves complex mechanisms [11,12]. Synovial inflammation appears to be one of the major contributors to pain and OA progression [11], with immunological mechanisms involving both innate and adaptive immunity considered to play a key role in driving inflammation and tissue destruction [12]. In addition, there is evidence that cartilage degeneration may be preceded by subchondral bone lesions (SBMLs), suggesting a key role for this process in the pathogenesis and progression of OA. SBMLs are associated with pain and activation of pro-inflammatory pathways that ultimately contribute to subchondral sclerosis and increased bone density. The subchondral bone undergoes abnormal remodeling at sites where released metalloproteinases degrade the extracellular matrix (ECM) and, consequently, the cartilage itself. This process contributes to the formation of subchondral cysts and osteophytes [13]. Collectively, these findings support the concept of OA as a complex, multifactorial joint disease driven by inflammatory and metabolic factors [12].

Subchondral bone remodeling is considered as a key component of OA pathogenesis and may compromise the integrity of the osteochondral unit. Negative impact on the bone structure is also a characteristic feature of RA [1,5,7]. Under normal physiological conditions, bone resorption and formation are maintained by a balance between osteoclast and osteoblast activity. In RA, this balance is disrupted, resulting in accelerated bone resorption and inadequate bone formation [14]. Several key cytokines involved in bone resorption, including receptor activator of nuclear factor kappa-B ligand (RANKL), a member of the tumor necrosis factor (TNF) superfamily, TNF-α, interleukin-1 (IL-1), IL-6, and IL-17, play important roles in osteoclast differentiation. Under the inflammatory conditions associated with RA, activated T lymphocytes and fibroblast-like synoviocytes (FLS) express high levels of RANKL, thereby promoting local bone destruction [3,14].

Multiple autoantigens have been characterized in patients with RA, including type II collagen (COL2) (as a main structural protein of articular cartilage), circulating serum proteins, enzymes, and others, along with autoantibodies directed against these antigens [2,15]. Elevated levels of antibodies have also been described in OA. These immunoglobulins are directed against various proteins, including aggrecan, fibronectin, proteoglycans, cartilage oligomeric matrix protein (COMP), as well as the native forms of type I and type II collagen. Autoantibodies against native collagen have been detected in both forms of arthritis, RA and OA [16]. In addition to antibodies against the native forms of proteins, patients with RA also develop humoral responses against post-translationally modified proteins [17]. A typical example are autoantibodies that recognize citrullinated proteins. Citrullination is a post-translational modification (PTM) that is important for certain aspects of protein function, but it also plays a role in autoimmune diseases such as RA. Citrullination is a process in which the amino acid arginine is converted into citrulline within the protein structure. This reaction is catalyzed by peptidyl arginine deiminase (PAD) enzymes [18]. Two PAD isoforms, PAD2 and PAD4, have been identified as being expressed during joint inflammation in patients with RA, and both enzymes can catalyze the citrullination of COL2 [19]. From an autoimmune perspective, PTMs such as citrullination can alter the structure of proteins, affect their charge and other important properties, or result in the generation of novel antigenic epitopes that may trigger an autoimmune response [17]. In RA, autoimmune responses can be directed against the citrullinated forms of various proteins, including vimentin, filaggrin, type II collagen, enolase, fibrinogen, histones, and others.

ACPAs are a highly characteristic marker of RA, demonstrating a specificity of more than 90%. Autoantibodies reactive against citrullinated COL2 have been detected in the serum and synovial fluid of patients with RA [18]. In the articular cartilage of patients with OA and RA, the inflammatory environment stimulates chondrocytes to secrete large amounts of matrix-degrading enzymes, including matrix metalloproteinases (MMPs), particularly MMP-1 and MMP-13, which contribute to the degradation of COL2 and development of potential anti-COL2 reactivity [2]. MMP-13 is considered a major enzyme involved in ECM degradation and plays a key role in the cleavage and degradation of type II collagen [20]. Citrullination has been shown to delay the degradation of modified collagen molecules by MMP-13, while ACPAs further inhibit MMP-13 activity at different cleavage sites, thereby promoting the generation of collagen fragments that differ from those produced under physiological conditions [19].

Autoantibodies, including ACPAs and anti-COL2 antibodies, have been analyzed in patients with RA and OA [10]. Although ACPAs are a key characteristic of RA, antibodies against COL2 may overlap between RA and OA [16]. The present study aimed to characterize the antibody reactivity against COL2 epitopes in patients with OA, with particular emphasis on immune responses against citrullinated COL2 epitopes, and to compare these responses with those observed in RA patients and non-arthritic controls. Part of the main goal of the study was to evaluate the extent of citrullination of blood plasma proteins and articular COL2 across the study groups and to determine whether citrullinated residues are present in normal hyaline cartilage. These investigations were undertaken to clarify the relationship between COL2 citrullination, anti-citrullinated COL2 immunity, and the potential contribution of altered citrullination to the pathogenesis of OA and RA.

## 2. Materials and Methods

### 2.1. Patients and Sample Collection

The present study involved 40 RA patients (mean age 58 ± 11 years; 29 women and 11 men), 30 OA patients (mean age 70 ± 10 years; 26 women and 4 men) and 23 control individuals with no clinical signs of rheumatic disease (mean age 54 ± 7 years, 15 women and 8 men). All subjects have signed informed consent for inclusion in the study. RA patients have been diagnosed more than a year prior participation in the study according to the American College of Rheumatology criteria [21] and had already received treatment (biologic or conventional disease modifying antirheumatic drug therapy). All of them were ACPA positive. OA patients had bilateral knee involvement and were radiologically graded according to the Kellgren–Lawrence system (grade 2–4; among them 15 patients had grade 2, 11 patients had grade 3, and 4 patients were classified with grade 4). All experiments were performed in concordance to the Declaration of Helsinki and approved by the Local Ethical Committee at Paisii Hilendarski University of Plovdiv, Bulgaria (Protocol No. 7 from 10 June 2024).

Peripheral blood was collected from the cubital vein of the patients and control individuals into BD Vacutainer^®^ K2 EDTA tubes (Becton, Dickinson and Company, Oakville, ON, Canada). Then, the samples were centrifuged for 20 min at 1000 rpm at room temperature (RT) and the plasma was collected in a sterile cryogenic vials. The samples were stored at −20 °C until analyzed. All samples were coded and assayed in a blinded way.

### 2.2. COL2 Preparation and COL2 Peptides

COL2 was prepared from human hip joint cartilage, which was obtained from subjects without clinical signs of rheumatic disease undergoing replacement surgery after an injury. COL2 from RA and OA patients was prepared from knee joint cartilage obtained during arthroplasty. All cartilage samples were treated with 4 M guanidinium chloride to remove proteoglycans. Collagen was subsequently solubilized by pepsin digestion in 0.5 M acetic acid. NaCl was added to a final concentration of 2 M to precipitate the solubilized collagen, which followed by dialysis against a neutral buffer. The collagen was then purified by DEAE-Sepharose CL-6B ion-exchange chromatography. The minor cartilage collagens were precipitated at a final concentration of 0.7 M NaCl in 0.5 M acetic acid. Type I collagen was removed from by precipitation at 2.5 M NaCl in neutral buffer. The purity of the obtained COL2 samples was analysed by SDS PAGE (Supplementary Figure S1).

Four synthetic COL2 peptides corresponding to amino acid sequence COL2 358–369 (GARGLTGRPGDA; C1 epitope), COL2 358–369 (containing two citrulline residues: GACitGLTGCitPGDA), COL2 494–504 (LVGPRGERGFP; U1 epitope) and COL2 551–564 (MPGERGAAGIAGPK; J1 epitope) (all from Schafer-N, Copenhagen, Denmark) were used in the present study and denoted as COL2 (358–369), citCOL2 (358–369), COL2 (494–504), COL2 (551–564), respectively. All peptides and purified collagen samples were dissolved in 0.1 M acetic acid at concentration 5 mg/mL and stored at −20 °C and 4 °C, respectively until used.

### 2.3. Measurement of Anti-COL2 Antibodies and COL2 Epitope-Specific Antibodies

ELISA plates (Corning Inc., Corning, NY, USA) were coated with 20 µg/mL human COL2 or 5 µg/mL COL2 peptides overnight at 4 °C. Unspecific binding was blocked by incubation with phosphate buffer (PBS) containing 5% fetal bovine serum (Merck KGaA, Darmstadt, Germany) for 1 h at RT. Then the plates were washed three times with ELISA buffer (PBS supplemented with 0.05% Tween 20) and blood plasma samples in 1/10 dilution were added to the plates and incubated for 2 h at RT. The samples were coded and tested in a blinded manner. Subsequently, the plates were washed four times with ELISA buffer and 1 µg/ml goat anti-human IgA/IgG/IgM-HRP (Novus Biologicals, Centennial, CO, USA) solution was pipetted in all test-wells followed by incubation for 1 h at RT. After that, the plates were washed five times with ELISA buffer and peroxidase substrate solution was added to each well (ABTS, Merck KGaA, Darmstadt, Germany). As a result, color reaction developed in 5 to 15 min and absorbance at 405 nm wavelength was detected using SpectraMax i3x spectrophotometer (Molecular Devices, San Jose, CA, USA).

### 2.4. Evaluation of Anti-Cyclic Citrullinated Peptides (CCP) Responses

Anti-cyclic citrullinated vimentin peptides IgG antibodies were measured by ELISA using the anti-CCP hs^®^ test-system (ORGENTECH, Sebia, Lisses, France). The assay was performed in accordance with the manufacturer protocol and Alegria^®^ instrument (ORGENTECH, Sebia, Lisses, France) was used to detect and determine anti-CCP antibodies concentration in the analyzed samples.

### 2.5. Detection of Citrullinated Proteins

To determine the relative extent of citrullination ELISA plates (Corning Inc., Corning, NY, USA) were coated overnight at 4 °C with 50 µg/mL COL2 samples or 50 µL/well blood plasma. A negative control coated with 5 µg/mL non-citrullinated COL2 peptides was assayed in all experiments as well as a positive control coated with 50 µg/mL PAD-treated fibrin. After coating the plates were incubated at RT for 1 h with blocking solution (PBS + 3% non-fat dry milk). Subsequently, the plates were washed three times with ELISA buffer. Then, 1 µg/mL FITC-conjugated anti-citrulline mouse monoclonal antibody (clone 6C2.1 (SAB5202304); Sigma-Aldrich, Saint Louis, MO, USA) was added to the plates and incubated for 1 h at RT. The plates were washed 5 times with ELISA buffer and fluorescence intensity was measured (excitation 488 nm/emission 520 nm) with SpectraMax i3x spectrophotometer (Molecular Devices, San Jose, CA, USA). All samples were analyzed in duplicates.

### 2.6. Immunostaining of Cartilage Specimens

Normal hyaline cartilage biopsies were obtained during nose plastic surgery of six patients that do not suffer arthritic disorder (mean age 22 ± 6 years; 4 women and 2 men). All samples were fixed in 10% buffered formaldehyde (pH 7.0) for 1 h at room temperature (RT) followed by overnight incubation at 4 °C. After fixation, the tissue samples were rinsed in tap water, dehydrated by incubation in graded ethanol series (50%, 70%, 80%, 95%, 100% ethanol) and embedded in paraffin wax. Sections with a thickness of 5–7 µm were obtained from all specimens using semi-automated rotary microtome (Leica RM 2125, RTS, Leica Microsystems, Wetzlar, Germany) and mounted on sterile glass slide. Ten paraffin sections from a different location on the paraffin block were obtained from each specimen. The mounted paraffin sections were treated with xylene for 5–10 min followed by incubation in 100% ethanol for 5–10 min. After that the mounted sections were washed in PBS. Washing with PBS containing 0.05% Tween20 was then performed. This was followed by blocking for 30 min with PBS containing 3% BSA and 0.05% Tween20. The specimens were rinsed in PBS and incubated for 1 h with anti-citrulline mouse monoclonal antibody conjugated with FITC (Sigma-Aldrich, Saint Louis, MO, USA) solution with dilution 1:50. An isotype control with mouse monoclonal antibody from the same isotype (IgG1) as the anti-citrulline antibody but different specificity was also analyzed. The slides incubated with fluorochrome-conjugated monoclonal antibodies were rinsed in PBS and analyzed on an epifluorescent microscope (Leica DM 2000 LED, Microsystems, Wetzlar, Germany) equipped with a digital camera (Leica K5C, Microsystems, Wetzlar, Germany). Images of the analyzed samples were obtained by specialized imaging software (LAS X 5.3.0.29166). Evaluations of immunostaining were performed in a blinded manner.

### 2.7. Statistics

Statistical analyses were performed with IBM SPSS Statistics (version 28.0, Armonk, NY, USA). Normality was assessed using the Shapiro–Wilk test for each measurement within each study group. As deviations from normality were observed, differences among the control, OA, and RA groups were evaluated using the Kruskal–Wallis test, followed by two-sided Dunn’s post hoc tests with Holm adjustment for multiple comparisons. Corrections for tied ranks were applied. For the primary analysis, *p*-values were adjusted across the three pairwise comparisons within each outcome. Sensitivity analyses additionally applied Holm correction across all pairwise comparisons and, separately, across the Kruskal–Wallis tests. Adjusted *p*-values < 0.05 were considered statistically significant.

To assess associations between anti-citCOL2 (358–369) and anti-CCP antibody levels within each study group, we used Spearman’s rank correlation coefficient (ρ). Associations between age and anti-COL2 antibody reactivity were assessed separately within the OA and RA groups. In the OA group, Spearman’s rank correlation was also used to examine the association between Kellgren–Lawrence grade and anti-citCOL2 (358–369) antibody levels. Correlation coefficients and their corresponding *p*-values were reported.

Reactivity to citrullinated and non-citrullinated COL2 (358–369) was compared within participants, separately in the OA (*n* = 30), RA (*n* = 40), and control (*n* = 23) groups. The paired differences were defined as Δ = anti-citCOL2 − anti-COL2. Negative values indicate lower reactivity to the citrullinated peptide.

The normality of the paired differences was assessed using the Shapiro–Wilk test. Within-group comparisons were evaluated using two-sided paired Student’s *t*-tests and Wilcoxon signed-rank tests. Exact two-sided Wilcoxon *p*-values were obtained from the sign-permutation distribution of the observed ranks, using average ranks for tied absolute differences and excluding zero differences. The Holm adjustment was applied to comparisons, separately for each testing method. Adjusted *p*-values < 0.05 were considered statistically significant.

Differences between the OA and RA groups were assessed using two-sided Dunn’s post hoc tests with Holm adjustment across the three pairwise comparisons within each outcome. We did not perform a formal equivalence test. Therefore, non-significant comparisons were interpreted as insufficient evidence of a difference rather than evidence of equivalence.

## 3. Results

### 3.1. Anti-COL2 and Anti-COL2 Epitope Antibody Reactivity in RA and OA Patients

**Figure 1:**
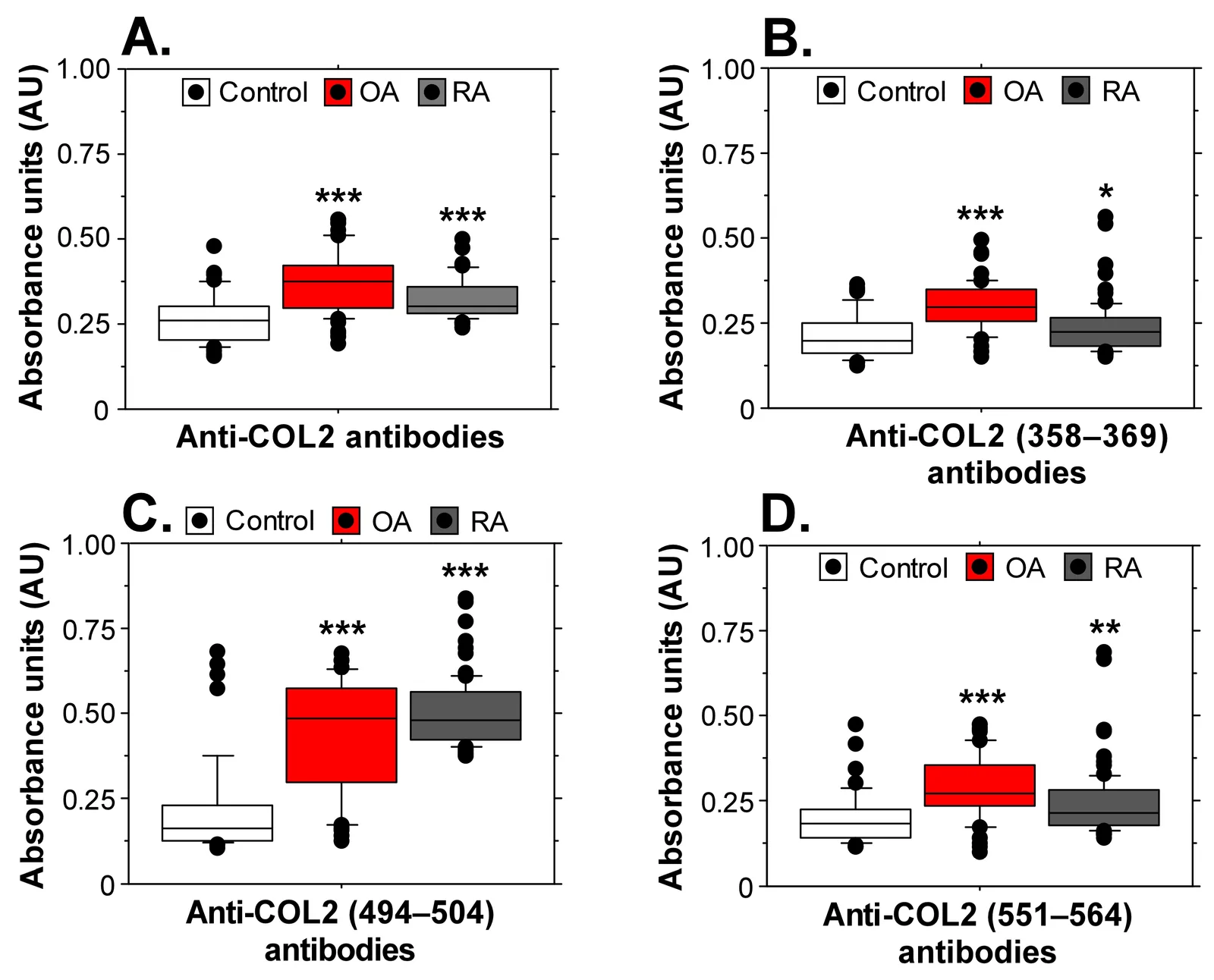
Levels of antibodies directed toward COL2 protein and COL2 epitopes. (**A**) Anti-COL2-specific antibodies; (**B**) Anti-COL2 (358–369)-specific antibodies; (**C**) Levels of antibodies that recognize COL2 (494–504) epitope; (**D**) Anti-COL2 (551–564) epitope-specific antibodies. Control (*n* = 23)—subjects without clinical signs of rheumatic disease; OA (*n* = 30)—osteoarthritis patients; RA (*n* = 40)—patients with rheumatoid arthritis. Boxes indicate Q1–Q3, horizontal lines indicate medians, and whiskers extend to the most extreme observed values within 1.5 times the interquartile range from the box edges. Quartiles were calculated by linear interpolation. Asterisks indicate two-sided Dunn comparisons with control, with Holm correction across all three pairwise comparisons within each panel: * *p* < 0.05, ** *p* < 0.01, *** *p* < 0.001. The asterisk in panel (**B**) is not significant under the sensitivity correction across all 12 comparisons (adjusted *p* = 0.0883).

To investigate and compare the anti-COL2 reactivity between the selected groups of OA and RA patients we analyzed the levels of IgG/A/M antibodies specific for human COL2 and COL2 epitopes defined in RA and mouse models of the disease. Significantly higher anti-COL2 reactivity was detected in OA patients compared to control subjects (Figure 1A). Interestingly, the levels of antibodies specific for the epitopes C1 (COL2 (358–369)), U1 (COL2 (494–504)), and J1 (COL2 (551–564)) were also markedly higher in OA patients compared to the control group (Figure 1B–D). Kruskal–Wallis tests showed significant group differences for anti-COL2 (H = 19.2841, *p* = 6.4941 × 10^−5^), anti-COL2 (358–369) (H = 21.7058, *p* = 1.9348 × 10^−5^), anti-COL2 (494–504) (H = 33.0988, *p* = 6.4966 × 10^−8^), and anti-COL2 (551–564) (H = 16.6637, *p* = 2.4073 × 10^−4^). With Holm adjustment within each outcome, both OA and RA had higher ranks than the control group for all four outcomes. OA had higher ranks than RA for anti-COL2, anti-COL2 (358–369), and anti-COL2 (551–564); the OA–RA comparison for anti-COL2 (494–504) was not significant (adjusted *p* = 0.1574). These data indicate that COL2-specific humoral responses are present in the studied OA patients from Bulgaria.

All four tests remained significant after Holm correction across outcomes. The control–RA comparison for anti-COL2 (358–369) was significant in the primary analysis (adjusted *p* = 0.0442) but not after correction across all 12 comparisons (adjusted *p* = 0.0883). All other pairwise significance conclusions at the 0.05 level were unchanged.

**Figure 2:**
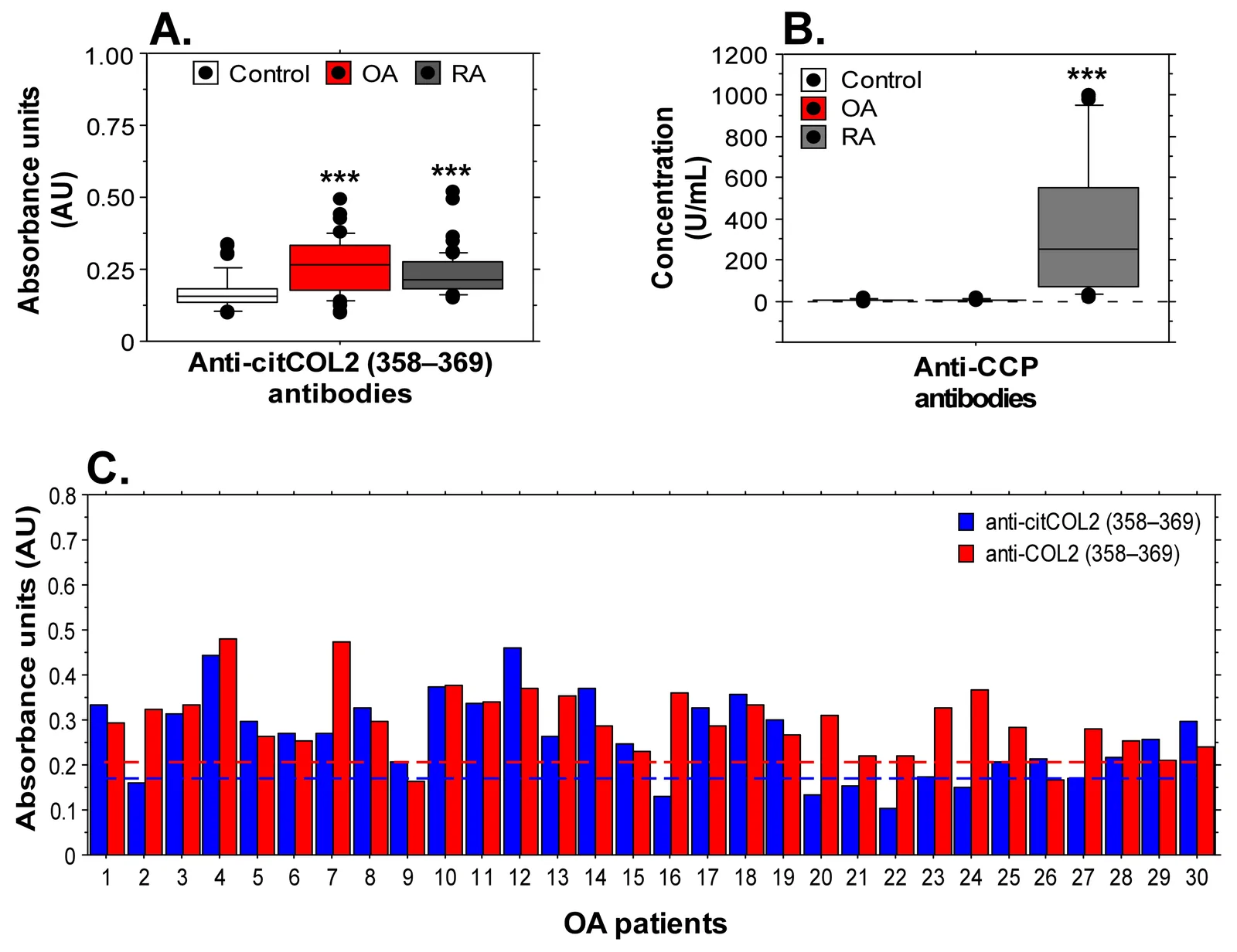
Antibody responses against citrullinated COL2 (358–369) epitope (**A**) and cyclic citrullinated vimentin peptides (**B**). Control—*n* = 23; OA—*n* = 30; RA—*n* = 40. Boxes represent the interquartile range (Q1–Q3), horizontal lines indicate medians, and whiskers extend to the most extreme observed values within 1.5 × IQR of the box edges. Asterisks indicate comparisons with the control group using two-sided Dunn tests with Holm correction across all three pairwise comparisons within each panel: *** *p* < 0.001. The dashed line in panel B marks zero (the displayed negative axis margin is for visual separation only). The left and the middle line in the same panel represent the control and the OA group, respectively. Plot (**C**) presents comparison of anti-COL2 (358–369) and anti-citCOL2 (358–369) antibody levels for each individual sample from the OA patients group. The blue horizontal line indicates the baseline level for anti-citCOL2 (358–369) antibody levels. The red horizontal line indicates the baseline level for anti-COL2 (358–369) antibody levels.

In addition to humoral responses against native collagen epitopes antibodies that recognize citrullinated C1 epitopes were analyzed. This evaluation aimed to determine potential immunity against citrullinated COL2 in the studied OA group and its possible association with anti-CCP titers specific for RA patients. As shown on Figure 2 concordant with the elevated levels of anti-COL2 unmodified epitopes antibodies OA patients showed significant response against the citrullinated C1 epitope (Figure 2A). Anti-citCOL2 (358–369) antibody measurements differed significantly among the three groups (Kruskal–Wallis H = 17.6674, df = 2, *p* = 1.4574 × 10^−4^). Dunn’s tests with Holm correction within this outcome showed higher ranks in OA and RA than in control (adjusted *p* = 1.7782 × 10^−4^ and 1.3421 × 10^−3^, respectively), with no significant difference between OA and RA (adjusted *p* = 0.1772). Anti-CCP measurements also differed among groups (H = 62.5260, df = 2, *p* = 2.6464 × 10^−14^). RA had higher ranks than both control and OA (adjusted *p* = 8.9714 × 10^−12^ and 5.3188 × 10^−9^, respectively), whereas the control–OA comparison was not significant (adjusted *p* = 0.3136). All significance conclusions were unchanged after Holm correction across the six pairwise comparisons.

**Figure 3:**
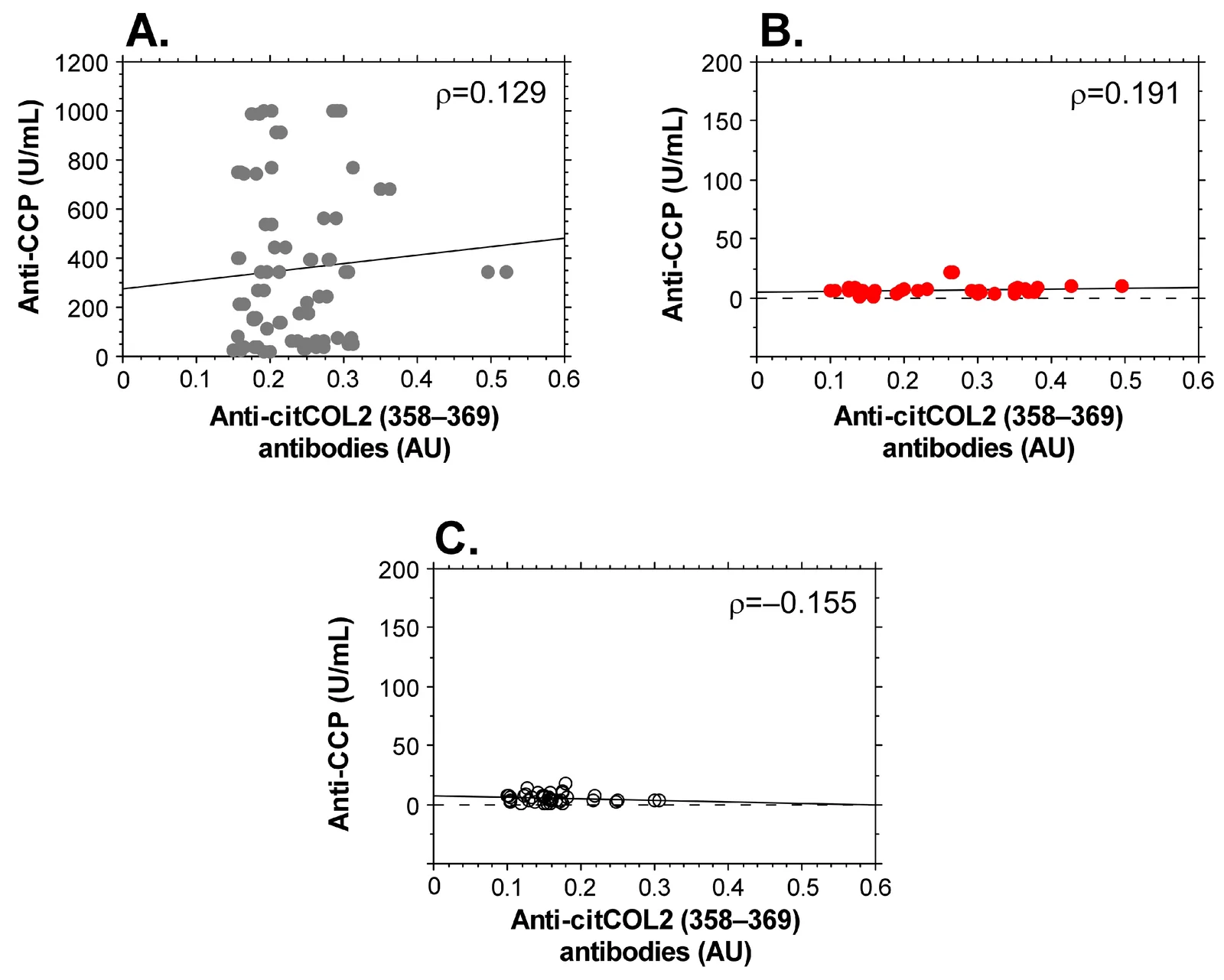
Analyses of the correlation between anti-CCP and anti-citCOL2 (358–369) antibody levels in RA patients (**A**), OA patients (**B**) and control subjects (**C**) ρ—Spearman rank correlation coefficient.

Despite the determined negative response against cyclic citrullinated vimentin peptides (Figure 2B) OA patients’ plasma contained markedly higher compared to the control group levels of anti-citCOL2 antibodies. These results suggest presence of anti-citrullinated COL2 immunity in both patients’ groups. Calculation of Spearman’s correlation that showed no significant correlation between anti-citCOL2 antibodies and anti-CCP antibody levels in RA (*p* = 0.2543) and OA patients (*p* = 0.2766) (Figure 3). In addition, marked correlation between patient age and anti-COL2 responses (Spearman rank correlation coefficient for OA ρ = 0.147, RA group—ρ = −0.234) was not observed. The radiographic scores of OA patients, assessed according to the Kellgren–Lawrence classification system, were also not significantly correlated with anti-citCOL2 antibody titers, as determined by Spearman’s correlation analysis (ρ = 0.003, *p* = 0.4495).

To investigate whether citrullination enhances COL2 (358–369) epitope recognition we compared the anti-COL2 (358–369) and anti-citCOL2 (358–369) antibody levels for each individual sample in the OA and RA group (Figure 2C; Supplementary Figure S2A,B). We did not observe predominant pattern of antibody response reactivity. 14 patients showed higher magnitude of individual antibody response against the citrullinated epitope compared to the native COL2 (358–369) while for 14 other patients the results were opposite (Figure 2C). 2 patients showed similar levels of anti-COL2 (358–369) and anti-citCOL2 (358–369) antibodies. Individual comparisons within the group of RA patients indicated that 15 subjects had higher antibody levels against the COL2 (358–369) and 13 patients showed higher anti-citCOL2 (358–369) responses. 12 RA patients showed similar levels of anti-COL2 (358–369) and anti-citCOL2 (358–369) antibodies (Supplementary Figure S2A).

Paired comparisons revealed lower reactivity to citrullinated COL2 (358–369) than to the corresponding non-citrullinated peptide in controls (mean paired difference, −0.0335; 95% CI, −0.0579 to −0.0091) (Supplementary Figure S2C). This difference remained statistically significant after Holm adjustment using both the paired t-test (adjusted *p* = 0.0278) and the Wilcoxon signed-rank test (adjusted *p* = 0.0326). No significant difference was detected in the RA group (adjusted *p* = 0.5051 and 0.4574, respectively). In the OA group, the lower mean anti-citCOL2 value was nominally significant using the unadjusted paired *t*-test (*p* = 0.0432), but did not remain significant after Holm adjustment (adjusted *p* = 0.0864) and was not supported by the Wilcoxon signed-rank test (adjusted *p* = 0.3444). Thus, these data did not definitively determine if epitope citrullination enhances its recognition.

### 3.2. RA and OA Patients Show Different Level of Citrullination of Joint COL2 and Blood Plasma Proteins

**Figure 4:**
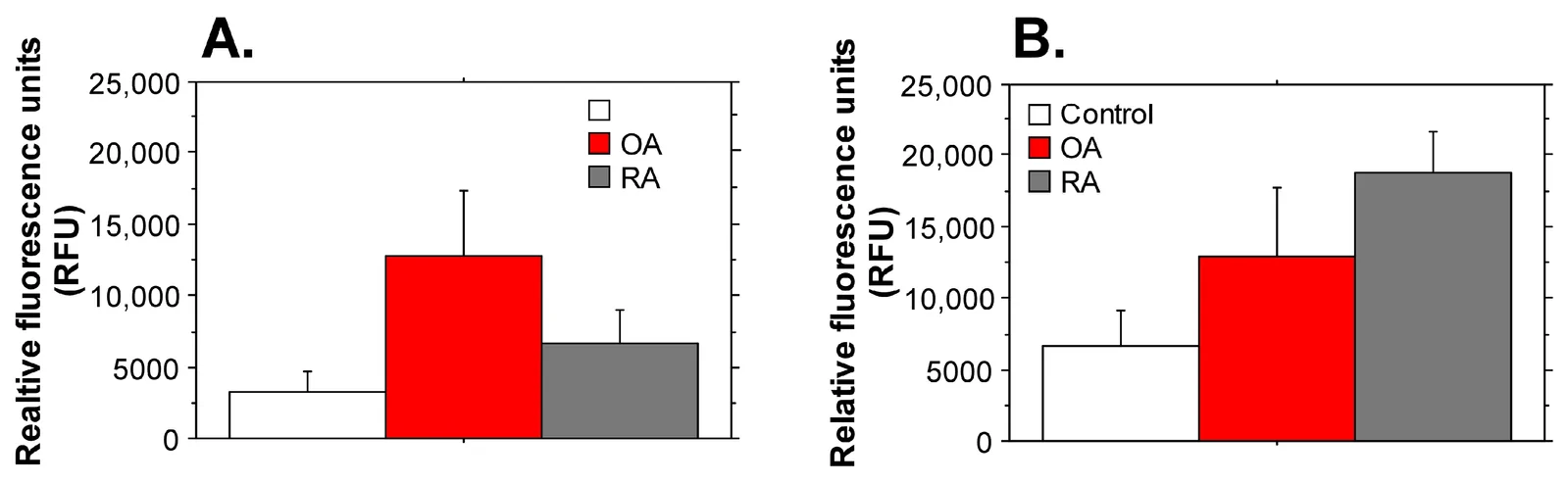
Relative levels of anti-citrulline immunoreactivity against COL2 (**A**) and blood plasma proteins (**B**). Data represent ± standard error of the mean (SEM). (**A**) Control *n* = 5, OA *n* = 3, and RA *n* = 6; (**B**) Control *n* = 5, OA *n* = 5, and RA *n* = 5. The repeated measurements shown in the figure represent independent biological samples. Group differences were evaluated using tie-corrected Kruskal–Wallis tests ((**A**): H = 2.7900, *p* = 0.2478; (**B**): H = 4.8200, *p* = 0.0898). Exploratory two-sided Dunn tests with Holm correction across three comparisons within each panel identified no statistically significant pairwise differences.

Based on the determined elevated levels of anti-citCOL2 antibodies in both groups with arthritic disorder and to further investigate the significance of citrullination process we performed ELISA assays directed to evaluation of the relative anti-citrulline immunoreactivity against COL2 purified from normal (non-arthritic), OA and RA articular cartilage as well as citrullination of blood plasma proteins from subjects without arthritic disorder, patients with OA and patients with RA (Figure 4). We found that relative COL2 citrullination was numerically higher in OA than in control, but the overall group difference was not statistically significant (Kruskal–Wallis H(2) = 2.7900, *p* = 0.2478; permutation *p* = 0.2523) (Figure 4A). Plasma protein citrullination was numerically highest in RA, but the overall difference was also not significant (H(2) = 4.8200, *p* = 0.0898; permutation *p* = 0.0886) (Figure 4B). No exploratory pairwise comparison was significant after Holm correction. In particular, the adjusted *p*-values were 0.2846 for control–OA COL2 and 0.0851 for control–RA plasma proteins. Our findings do not establish equivalence between groups, given the small number of samples and substantial variability. The determined tendency for higher relative anti-citrulline immunoreactivity in the OA and RA group compared to the control lead to the hypothesis for altered citrullination profile during both OA and RA pathogenesis. This was evident not only on the level of joint COL2 but also for blood plasma proteins.

### 3.3. Citrulline Amino Acid Residues Are Present in Normal Hyaline Cartilage of Individuals Without RA or OA

**Figure 5:**
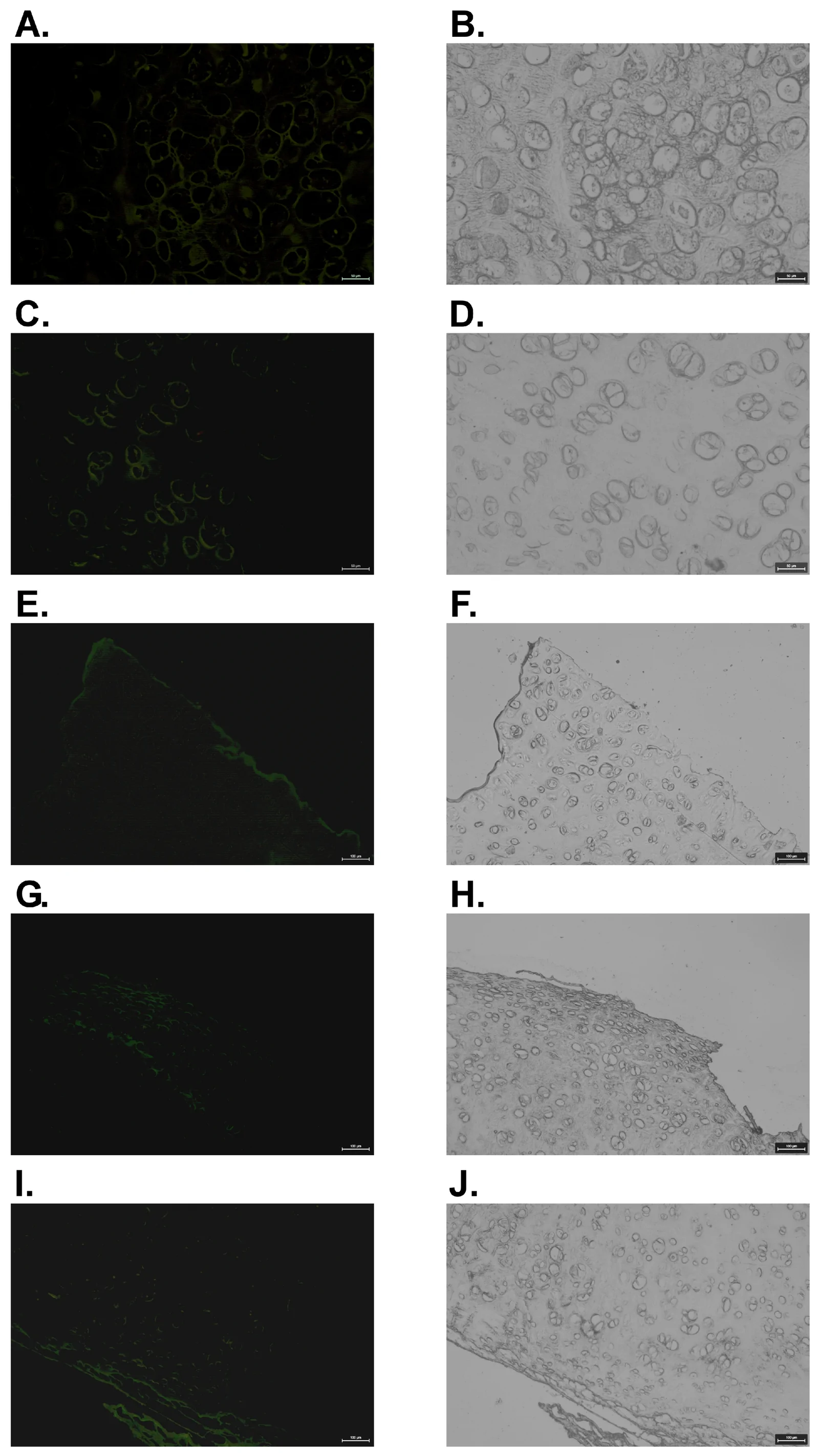
Microscopic observations of hyaline cartilage: fluorescent staining of hyaline cartilage (**A**,**C**,**E**,**G**,**I**) and corresponding bright field images (**B**,**D**,**F**,**H**,**J**). The markers on pictures (**A**–**D**) indicate 50 µm. The markers on pictures (**E**–**J**) indicate 100 µm.

The different levels of citrullination between non-arthritic controls and OA or RA patients leads to the question whether citrulline amino acid residues are present in the healthy cartilage. To address this point we performed immunofluorescence staining of normal hyaline cartilage using anti-citrulline antibodies. Samples obtained from 6 individuals were analyzed and all of them showed positive staining with anti-citrulline antibodies. Figure 5 illustrates the specific mode of the observed staining that indicated presence of citrulline in the perichondrium and the extracellular matrix surrounding chondrocytes. Although this observation was made in hyaline cartilage from a different anatomical site, it indicates that citrullinated proteins may be detectable under physiological conditions. It also raises the possibility that differences in citrullination profiles may be associated with disease-related changes and anti-citrulline immune responses; however, the nature and significance of this potential association remain to be determined.

## 4. Discussion

Citrullination is a hallmark of certain diseases, particularly rheumatoid arthritis, but it is clearly not restricted to pathological conditions. This post-translational modification (PTM) is, in fact, a physiological process in which the amino acid arginine is converted into citrulline through the enzymatic activity of peptidyl arginine deiminases (PADs). This irreversible PTM alters the charge and molecular mass of proteins, potentially affecting their structure and biological function. Several PAD isoforms (PAD1–PAD4 and PAD6) have been identified in different tissues, where they participate in diverse physiological processes, including apoptosis, gene regulation, and immune responses [22]. Furthermore, a large number of citrullinated proteins have also been detected in tissues from healthy individuals. In their review, Fert-Bober et al. reported that mass spectrometry analysis identified more than 200 citrullinated proteins in healthy human tissues, collectively referred to as the normal citrullinome, many of which are also found in patients with rheumatoid arthritis [23]. The presence of a large number of citrullinated proteins in various tissues from healthy individuals suggests that, theoretically, citrullination may also occur in articular cartilage under physiological conditions, and that citrullinated residues may be present within the structure of native type II collagen in healthy individuals.

Type II collagen is a major structural protein of articular cartilage and plays an important role in maintaining the extracellular matrix [16]. In an interesting study, Haag et al. demonstrated that COL2 can undergo citrullination while retaining its native triple-helical conformation following in vitro treatment with PAD2 enzymes at room temperature and at 37 °C. Autoantibodies against two epitopes located within the triple-helical molecule of type II collagen have been detected in patients with rheumatoid arthritis [24]. Furthermore, Glant et al. demonstrated that citrullinated protein structures are indeed present in normal human cartilage which was confirmed by our studies on normal hyaline cartilage. They identified citrullinated aggrecan, a major proteoglycan of the cartilage extracellular matrix, in both normal human cartilage and cartilage from patients with osteoarthritis (OA) and rheumatoid arthritis (RA) [25]. Sera from ACPA-positive RA patients reacted with PG purified from normal human cartilage specimens. PG fragments (mainly those containing the G1 domain) from OA or RA cartilage extracts were recognized by ACPA-positive sera but not by serum from ACPA-negative individuals. ACPA+ sera also reacted with in vitro citrullinated rhG1 and G3 domain-containing fragment(s) of PG. In their study, Li et al. reported that autoantibodies present in the sera of a subset of patients with RA and OA can react with both arthritic and healthy cartilage [10].

Despite the available evidence for physiological citrullination in articular cartilage from healthy individuals, the literature lacks convincing evidence regarding the presence of citrulline residues in, and citrullination of type II collagen in healthy individuals. Here, we present evidence that COL2 purified from non-arthritic joint and normal hyaline cartilage contain citrulline amino acid residues.

Physiological citrullination is a normal, tightly regulated post-translational modification that occurs transiently in specific tissues and cellular processes, with controlled peptidylarginine deiminase (PAD) activity and subsequent removal or turnover of citrullinated proteins [26]. In contrast, pathological citrullination is characterized by excessive, dysregulated, or persistent PAD activity, abnormal accumulation of citrullinated proteins, and their presence in inflammatory or damaged tissues. Pathological citrullination may also generate neoantigens that trigger anti-citrullinated protein antibodies (ACPAs), contributing to autoimmune inflammation, particularly in rheumatoid arthritis [27]. It is likely that the regulation of citrullination processes is severely impaired in RA, as increased citrullination has been observed compared with the normal citrullinome. In addition to the citrullinated proteins that are also found in healthy individuals, large amounts of intracellular and extracellular citrullinated proteins have been identified in the joints of patients with RA. These include enzymes, ribonucleoproteins, structural proteins, and others [23]. One of the structural proteins that undergoes deimination by PAD enzymes in RA is type II collagen. This is supported by the presence of autoantibodies against citrullinated COL2, as well as the detection of citrullinated COL2 fragments in the synovial fluid of patients with RA [28].

The inflamed synovium plays an important role in the destruction of articular cartilage through the secretion of pro-inflammatory mediators [8]. Activated synovial cells (particularly fibroblast-like synoviocytes—FLS), contribute to the degradation of articular cartilage. In RA, this process is closely associated with the inflammatory environment, in which FLS and synovial tissue macrophages (STMs) produce large amounts of enzymes that degrade articular cartilage and the underlying bone tissue, as well as cytokines that promote the recruitment and activation of immune cells, including tumor necrosis factor-alpha (TNF-α), interleukin (IL)-1β, and IL-6. Notably, the enzymes that ultimately mediate the degradation of the extracellular matrix show substantial overlap between OA and RA, including numerous matrix metalloproteinases (MMPs) and cathepsins [2]. Furthermore, activated macrophages in the synovial fluid of patients with OA also release pro-inflammatory cytokines, such as TNF-α and IL-1β [12]. It appears that citrullination plays an important role in synovial inflammation, cartilage degradation, and bone damage in the pathogenesis of RA, whereas the role of this PTM in OA remains not fully understood. The preliminary numerical observations for increased level of citrullination of OA COL2 determined by our studies suggest that the processes of destruction of normal joint cartilage could be a source of citrullinated antigens that may be involved in the disease progression, which would explain the detected immune reactivity against citrullinated COL2 in OA patients. Although the OA-derived COL2 preparations showed higher anti-citrulline immunoreactivity, the differences were not statistically significant. Given the small sample sizes and variability, this observation should be considered preliminary and does not establish a disease-associated increase in COL2 citrullination. Conversely, the absence of statistically significant differences does not demonstrate equivalence among the groups.

Purified antibodies from patients with RA directed against two citrullinated epitopes of COL2 can bind directly to articular cartilage. This finding suggests a potential destructive role of ACPAs in cartilage tissue, particularly against COL2, which is the major structural protein of cartilage [24].

Synovial inflammation is the main trigger of bone damage in RA [29], while osteoclasts possess the unique ability to resorb bone tissue, leading to bone loss [30]. The production of pro-inflammatory cytokines and RANKL, as well as the presence of ACPAs, stimulates osteoclast differentiation, thereby disrupting the balance between bone resorption and bone formation. This ultimately leads to periarticular bone erosions, a characteristic feature of RA [29].

As in RA, although to a different extent, immune cells are involved in osteoclastogenesis in OA through the secretion of RANKL [31]. All components of the osteochondral unit in OA joints are structurally and functionally affected due to their close interaction. These components include articular cartilage, calcified cartilage, and subchondral bone [32]. Osteoclasts can penetrate the osteochondral junction and promote the ingrowth of blood vessels and nerves into the deeper layers of articular cartilage. Following cartilage loss, cytokines from the synovial fluid can infiltrate the subchondral bone and affect the various cell populations residing there [31]. A characteristic feature of OA is subchondral sclerosis, in which increased bone remodeling is observed during the early stages of the disease and is associated with increased bone loss. This is followed by slower bone remodeling, ultimately leading to increased bone density in the later stages of OA progression [33]. These findings indicate that OA is not solely a disease of the cartilage but affects all surrounding joint structures. In a study involving 415 patients with RA and 304 patients with OA, autoantibodies against ACC10 (the 10 most frequently recognized citrullinated epitopes of type II collagen) were detected in 83.5% of patients with RA and 14.8% of patients with OA [10]. These findings indicate that antibodies against citrullinated COL2 are not restricted to RA; however, their role in the pathogenesis of OA remains unclear.

Several limitations should be considered when interpreting the findings of the present study. Our experiments primarily assessed the presence and overall extent of citrullination rather than providing a comprehensive characterization of individual citrullinated residues or their precise molecular locations within COL2 and other proteins. The preliminary numerical observations for increased citrullination of OA and RA COL2 and plasma proteins may contribute to the generation of autoimmune responses; alternatively, enhanced citrullination could occur secondary to inflammation, tissue injury, or alterations in the local cellular environment. The present data therefore demonstrate an association between altered citrullination and arthritic disease but do not establish whether these changes represent initiating pathogenic events or downstream consequences of disease. Consequently, it remains unclear whether particular patterns of citrullination are associated with OA or RA pathogenesis.

A limitation of this study is the difference in age distribution between the study groups. The OA group was older on average than both the RA and control groups, and although within-group analyses were performed to assess the association with age, residual confounding due to between-group age differences cannot be completely excluded. Therefore, the observed differences between groups should be interpreted with consideration of the potential influence of age. Another limitation of the present study is that detailed information regarding patients’ pharmacological treatment regimens prior to sample collection was not systematically collected. In particular, patients with RA had received conventional and/or biologic DMARDs, while patients with OA had received analgesic or other symptomatic treatments. Therefore, the potential effects of prior medication use on anti-citCOL2 antibody concentrations and related immune responses could not be evaluated. Considering the relatively small size of the studied OA patient cohort and the lack of longitudinal assessment it cannot be determined whether anti-citCOL2 antibodies represent a distinct autoimmune response or arise as a consequence of tissue-specific exposure to citrullinated collagen following cartilage damage.

An additional consideration is the different anatomical origin of the cartilage samples used for COL2 isolation. Control COL2 was obtained from hip joint cartilage, whereas COL2 from OA and RA patients was isolated from knee joint cartilage. Although articular cartilage from different joints may exhibit some variation in its molecular characteristics, the extent to which joint origin may have influenced the observed citrullination profiles or anti-citrulline immunoreactivity is uncertain. Therefore, a contribution of anatomical origin to the observed differences cannot be completely excluded. Since control and disease-derived COL2 were not obtained from the same joint, the potential effects of joint origin cannot be fully separated from disease-associated differences in the present study.

Finally, although the detection of citrullinated residues in healthy cartilage is an important finding, the study does not fully establish how physiological citrullination differs from the altered citrullination profile observed in OA and RA. Determining whether disease-associated changes involve increased abundance, altered localization, generation of novel epitopes, or changes in the accessibility of citrullinated residues will require further investigation.

## 5. Conclusions

The present study demonstrates that citrullination of collagen type II may be involved in both rheumatoid arthritis (RA) and osteoarthritis (OA), indicating that immunity against this post-translational modification could be associated with development of different arthritic disorders.

Our data showed that patients with OA have increased antibody reactivity to native COL2 and the tested COL2-derived peptides, including citCOL2 (358–369), compared with non-arthritic controls. These findings suggest the presence of humoral reactivity to a citrullinated COL2 epitope in OA. However, the accompanying reactivity to native COL2 epitopes and the individual native–citrullinated peptide comparisons do not conclude that this response is specifically driven by citrullination.

We detected anti-citrulline immunoreactivity in COL2 preparations from non-arthritic joints and in normal nasal hyaline cartilage, supporting the occurrence of citrullination outside clinically apparent arthritic disease. OA-derived COL2 preparations showed a tendency for higher anti-citrulline immunoreactivity, suggesting that citrullination of COL2 and the resulting immune response may have a previously underrecognized role in OA.

Overall, our findings are basis for further investigations of COL2-directed antibody responses and citrullination in OA. Larger studies with age- and anatomical-site-matched samples, better characterization of treatment exposure, and independent confirmation of COL2 citrullination are needed to clarify the biological significance of these observations and to clarify the relationship between citrullination, immune responses, and cartilage damage.

## Data Availability

The original contributions presented in this study are included in the article.

## Supplementary Materials

The following supporting information can be downloaded at: https://www.mdpi.com/article/10.3390/biology15201877/s1, Figure S1: SDS-PAGE analyses of COL2 samples. Representative images of COL2 samples from each experimental group; Figure S2: Paired reactivity to citrullinated and non-citrullinated COL2 (358–369) epitope; Figure S3: Controls for immunofluorescent staining of hyaline cartilage.

## Abbreviations

The following abbreviations are used in this manuscript:

ACPA	Anti-citrullinated protein antibodies
COL2	Collagen type II
ECM	Extracellular matrix
EDTA	Ethylenediaminetetraacetic acid
ELISA	Enzyme-linked immunosorbent assay
FBS	Fetal bovine serum
FITC	Fluorescein isothiocyanate
HRP	Horseradish peroxidase
Ig	Immunoglobulin
IL	Interleukin
MMP	Matrix metalloproteinase
OA	Osteoarthritis
PAD	Peptidyl arginine deiminase
PBS	Phosphate-buffered saline
PTM	Post-translational modification
RA	Rheumatoid arthritis
RANKL	Receptor Activator of Nuclear Factor-κB Ligand
RT	Room temperature
SDS-PAGE	Sodium Dodecyl Sulfate Polyacrylamide Gel Electrophoresis
TNF-α	Tumor necrosis factor alpha
