# Supplementary material for: Citrullination of Collagen Type II and Epitope-Specific Antibody Responses in Rheumatoid Arthritis and Osteoarthritis: A Comparative Analysis
Source: Biology (Basel). 2026 Oct 18;15(20):1877. doi: 10.3390/biology15201877 (PMC13701949; doi:10.3390/biology15201877)
Supplement: Supplementary file 1 [file biology-15-01877-s001.zip › biology-4586699-supplementary.pdf]

Supplementary Materials

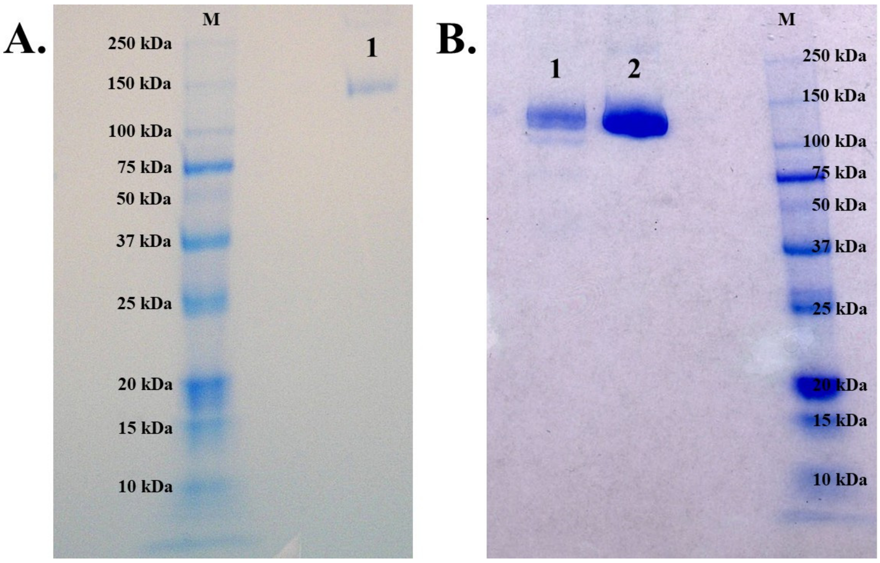

Figure S1. SDS-PAGE analyses of COL2 samples.
Representative images of COL2 samples from each experimental group. **A.** COL2 sample from control subject (lane 1). **B.** Sample from OA patient (lane 1) and RA patient (lane 2). Lane M - protein molecular weight markers.

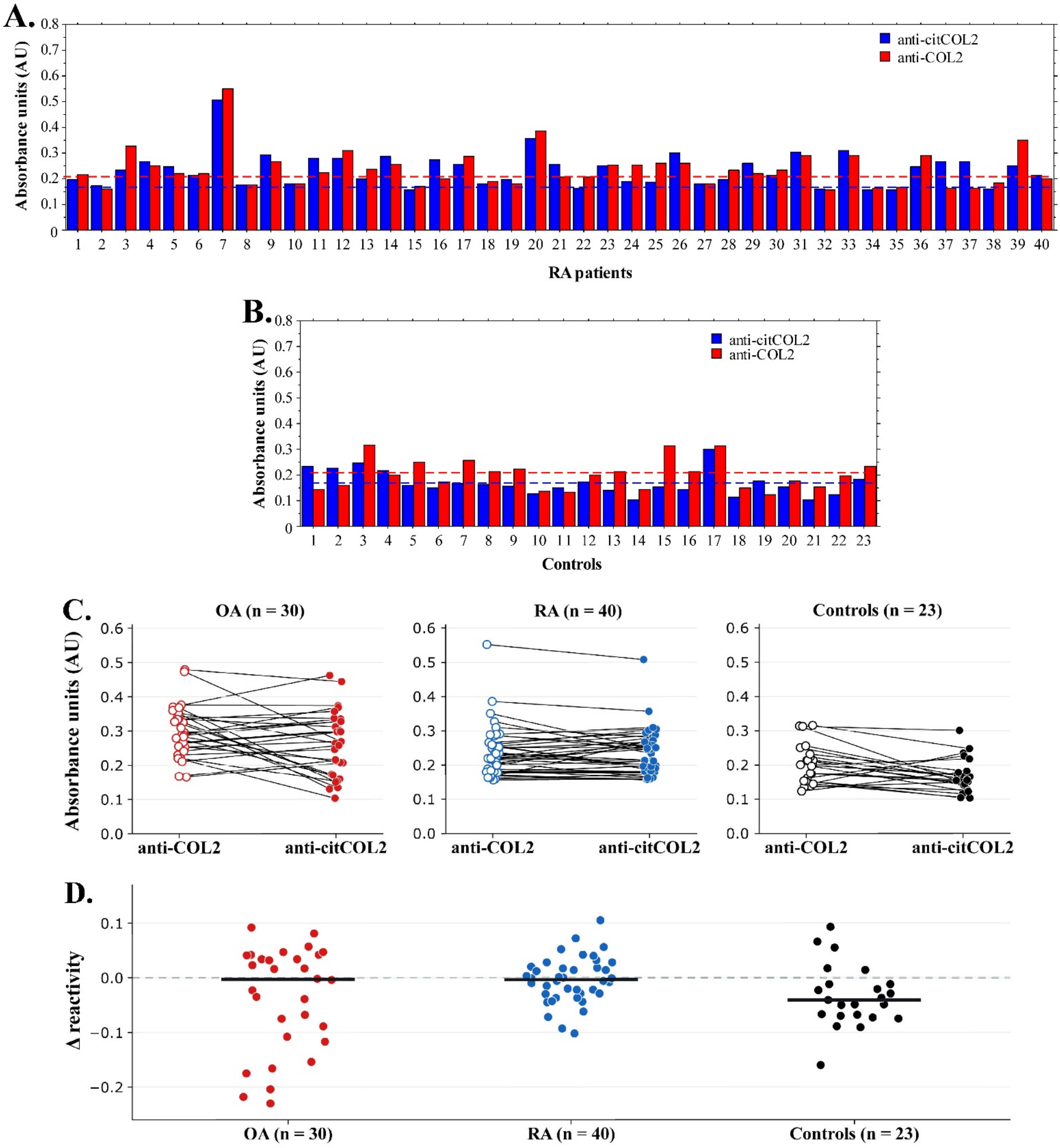

Figure S2. Paired reactivity to citrullinated and non-citrullinated COL2 (358–369) epitope.

A. RA patients group; **B.** control subjects. The blue horizontal line indicates the baseline level for anti-citCOL2 (358-369) antibody levels. The red horizontal line indicates the baseline level for anti-COL2 (358-369) antibody levels. **C.** Individual measurements in OA (n = 30), RA (n = 40), and controls (n = 23). Lines connect measurements from the same participant. **D.** Within-participant differences (Δ-reactivity), calculated as anti-citCOL2 (358–369) minus anti-COL2 (358–369). Each point represents one participant, and black horizontal bars indicate group medians of paired differences. The dashed line at zero denotes equal measured reactivity to the two peptides and does not represent a positivity cutoff. Negative values indicate lower reactivity to the citrullinated peptide. Adjusted p values: p = 0.3444 for OA; p = 0.4574 for RA; p = 0.0326 for controls.

Reported p-values are determined by two-sided Wilcoxon signed-rank tests, adjusted across the three within-group comparisons using the Holm method.

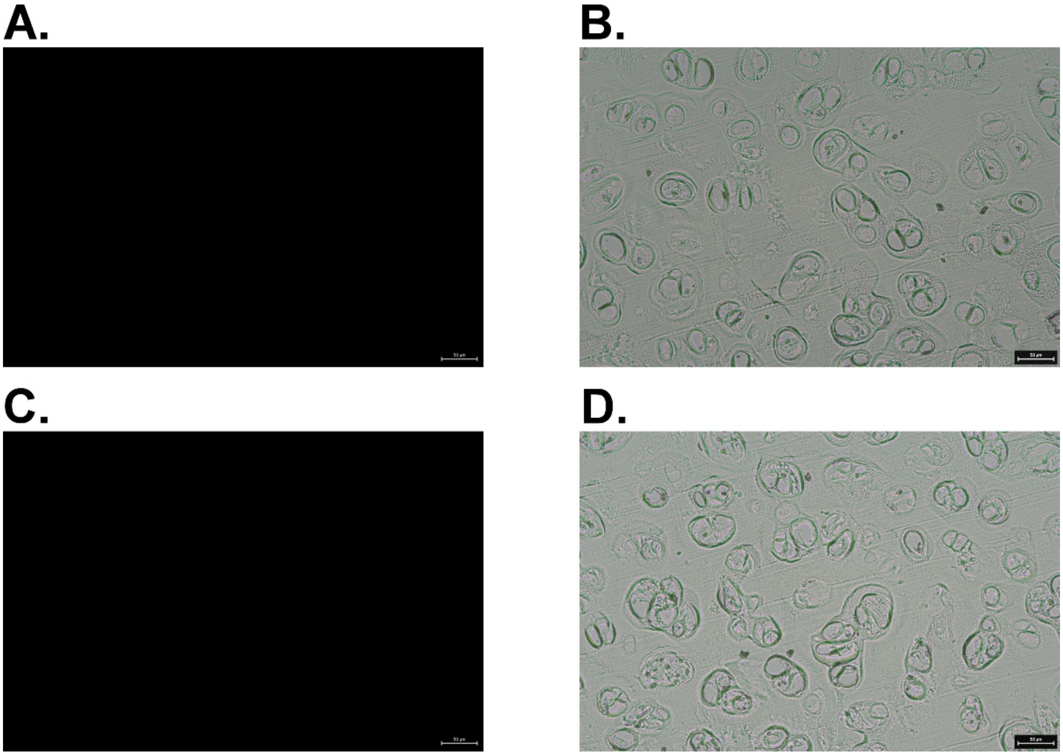

Figure S3. Controls for immunofluorescent staining of hyaline cartilage: isotype control (**A.**), unstained specimen (**B.**) and corresponding bright field images (**C.**, **D.**). The markers on the pictures indicate 50 μm.
